# Hydropower station scheduling with ship arrival prediction and energy storage

**DOI:** 10.1038/s41598-023-45995-3

**Published:** 2023-11-03

**Authors:** Enjiang Zhou, Xiao Liu, Zhihang Meng, Song Yu, Jinxiu Mei, Qiang Qu

**Affiliations:** 1 Guizhou Wujiang River Navigation Authority, Tongren, 565100 China; 2 College of Mathematics and Information Science, Hebei University, Baoding, 071002 China; 3 Shenzhen Institute of Advanced Technology, Chinese Academy of Sciences, Shenzhen, 518055 China; 4 Guizhou Goupitan Navigation Authority, Yuqing, 563102 China; 5 Guizhou Zhongnan Transport Technology Co. Ltd., Guiyang, 550000 China

**Keywords:** Power stations, Computer science

## Abstract

Effectiveness improvement in power generation and navigation for grid-connected hydropower stations have emerged as a significant concern due to the challenges such as discrepancies between declared and actual ship arrival times, as well as unstable power generation. To address these issues, this paper proposes a multi-objective real-time scheduling model. The proposed model incorporates energy storage and ship arrival prediction. An energy storage mechanism is introduced to stabilize power generation by charging the power storage equipment during surplus generation and discharging it during periods of insufficient generation at the hydropower stations. To facilitate the scheduling with the eneragy storage mechanism, the arrival time of ships to the stations are predicted. We use the maximization of generation minus grid load demand and the maximization of navigability assurance rate as two objective functions in the scheduling process. The model uses the Non-Dominated Sorting Beluga Whale Optimization (NSBWO) algorithm to optimize and solve the real-time discharge flow scheduling of the hydropower stations in different time periods. The NSBWO algorithm combines the Elitist Non-Dominated Sorting Genetic Algorithm (NSGA-II) and the Beluga Whale Optimization (BWO). The experimental results show that the proposed method has advantages in predicting the expected arrival time of ships and scheduling the discharge flow. The prediction using XGBoost model reaches accuracy with more than 0.9, and the discharged flow obtained from scheduling meets the demand of hydropower stations grid load while also improves the navigation benefits. This study provides theoretical analysis with its practical applications in a real hyropower station as a case study for solving hydropower scheduling problems.

## Introduction

Building a new energy power system is one of the important ways to achieve the goal of carbon peaking and carbon neutrality^1^. In the process of power system transformation, new energy power represented by water conservancy and hydropower is incorporated into the power grid system in a high-speed and large-scale manner^2^. Under the new operating environment, grid-connection hydropower stations must undertake comprehensive utilization tasks such as power generation, shipping, and flood control^3^. Hydropower is a renewable energy source and the process of water circulation is continuous. The introduction of hydroelectricity in water distribution networks allows for the sustainable use of energy by utilizing the kinetic energy of water flow to generate electricity. This helps to promote the goal of sustainable development and reduce dependence on finite energy resources^4^. This is an important support for the construction of a new power system under the dual carbon goals and a reliable guarantee for the efficient use of water resources. At the same time, hydropower stations are facing both higher intensity and frequency of grid peaking and frequency regulation tasks. The requirements from both water conservancy and electric power generation also greatly limit the space for the optimization of scheduling operations of hydropower stations, easily causing problems such as unbalanced scheduling, inadequate benefits and poor ship navigation. How to better coordinate power scheduling and navigation quality, further improve the joint optimal scheduling benefits of hydropower stations and reduce the waiting time of ships passing through the gates are the key to ensure the effectiveness of the hydropower stations.

Optimal scheduling of hydropower stations with dual generation-navigation objectives is one of the classical problems in the direction of optimal scheduling of engineering systems. To solve this kind of problem, it is necessary to formulate an optimal scheduling problem into a multi-dimensional, nonlinear, multi-stage, strictly constrained optimization problem^5,6^. Under the condition of satisfying the constraints and the load demand of the power grid, guided by the methods and theories in the field of operation research, the scheduling strategy is used to obtain the scheduling rules with better shipping benefits.

The essence of optimal scheduling of hydropower stations for power generation and shipping is to build and solve mathematical models, which often consist of two parts, scheduling objectives and constraints. Mathematical models are generally divided into the following categories: (1) “water to determine electricity” with maximum generation capacity and maximum generation efficiency, (2) “electricity to determine water” with minimum water consumption and maximum end-of-period energy storage, (3) downstream ecological protection with minimal ecological impact as the objective function. The constraints are also very complex and diverse. The solutions are usually divided into two categories: mathematical programming and intelligent optimization algorithms. Mathematical programming mainly includes Linear Programming^7^, Nonlinear Programming^8^, Mixed Integer Programming^9^, and Dynamic Programming^10^. With the development of operation research, economics and new theories and the improvement of computational difficulty and computational accuracy of hydropower station scheduling, bionic intelligent optimization models based on Genetic Algorithm^11^, Particle Swarm Algorithm^12^, Ant Clony Optimization^13^ emerged.

Since the difference between the actual arrival time of ships and the declared arrival time might be large, it often increases the waiting time for the ship to pass the gate, making the scheduling effectiveness of the hydropower station low. Meanwhile, the power generation capacity of hydropower stations are not stable due to the restrictions on discharge flow. Currently, most of the existing studies focus on the selection and improvement of optimization strategies and optimization algorithms, without much research on the impact of real-time ship passage through the gates in hydropower stations on the optimization of hydropower stations scheduling. The role of energy storage devices in stabilizing power generation is also not considered in most of the existing studies.

Therefore, our study is to improve the operational effectiveness of the grid connected hydropower stations and to balance the benefits of shipping and power generation effectively. In the actual scheduling process we need to consider the difference between the actual arrival time of ships and the declared arrival time, as well as the shortage or surplus of power generation in hydropower stations. This paper proposes a new multi-objective real-time scheduling model to solve the joint scheduling problem of hydropower generation and shipping by using prediction algorithm, energy storage and intelligent optimization. We also apply the method in a real application of the Wujiang River as a case study. The main contribution of this paper is summarized as follows.

(i) Energy storage is introduced in the scheduling process of hydropower stations in order to stabilize the power generation. If the power generation during the scheduling time period is higher than the corresponding grid load demand, the energy storage device is charged, and conversely, if the power generation is insufficient, the storage device is discharged to ensure that the grid power load demand is satisfied.
(ii) The power generation during the scheduling time period is analyzed where a new objective function considering the energy storage is proposed. For the function, we consider the factors that might affect shipping and power generation effectiveness such as water level, flow rate and other constraints.
(iii) An improved multi-objective beluga optimization algorithm, namely NSBWO, is proposed to encode and schedule the solution for the discharge flow during the scheduling time of the hydropower stations.

The structured rest of the paper is as follows: “Related work”  discusses existing studies regarding the scheduling of hydropower stations. “Ship estimated arrival time prediction”  presents the XGBoost model to predict the expected arrival time of ships. “The proposed real-time scheduling model”  analyzes the constraints in the scheduling process and introduces the improved NSBWO scheduling model. “Experimental discussion”  evaluates our model with multiple metrics, providing the insights of the proposed method. Finally, “Conclusion”   concludes the paper with future work.

## Related Work

In recent years, quite a few studies have been proposed on the real-time scheduling of hydropower stations. For example, Yang et al.^14^ explored the particle swarm optimization algorithm for hierarchical multi-objective optimization problems and its application to the optimal operation of hydropower stations, and optimized the scheduling with the two objectives of maximizing the peak energy efficiency and maximizing the power generation of hydropower stations. Hidalgo et al.^15^ combined the evolutionary and the gradient algorithms to propose a model for optimizing the short-term operation of hydropower stations. Jia et al.^16^ proposed a scheduling model considering both daily maximum generation and navigation demand, and obtained the optimal solution by genetic algorithm. Meng et al.^17^ proposed an improved multi-objective labeled cuckoo search algorithm based on a constrained transformation population initialization strategy to solve the trade-off between water and energy in the context of the Xiaolangdi and Xi’an terrace hydropower stations in the lower Yellow River in China. Fang et al.^18^ proposed a scheduling model for reservoir ecological protection and power generation maximization in the context of the Minjiang River Shuikou Hydropower Stations in China, and solved it using an improved multi-objective particle swarm optimization algorithm. Marcelino et al.^19^ proposed an efficient mathematical model for hydropower stations scheduling that is solved using a coral optimization algorithm with different search operators in a single population. Feng et al.^20^ proposed a new multi-objective particle swarm optimization algorithm with the goal of maximizing power generation. The method introduces recursive mapping, which makes the initial population evenly distributed in the problem space, and the inertia weight and learning coefficient change dynamically with the back generation. Marcelino et al.^21^ used a multi-objective evolutionary group hybrid algorithm to solve the short-term hydropower unit configuration problem and compared it with other optimization methods. Chen et al.^22^ developed a coordination model of hydropower and ecological flow using three models NSGA-II, NSGA-III and RVEA. After comparison, the NSGA-III model was found to have certain advantages. Wang et al.^23^ took the minimum load peak-to-valley difference of multiple power grids as the objective function. Transform the original nonlinear non-convex model into a standard mixed-integer linear programming (MILP) formulation through several linearization strategies.

Most of the existing studies aimed at maximizing generation capacity and efficiency through static scheduling during the scheduling time period. Few considers the hydropower stations that have both shipping and power generation demands, and the application of energy storage combined with hydropower generation in stabilizing grid peaking.

## Ship estimated arrival time prediction

### Research purpose

In order to realize the combination mode of “weekly sluice plan declaration + daily sluice plan control + sluice real-time scheduling” of the Wujiang Silin Hydropower Station in Guizhou province of China. The scheduling center needs to use technical means such as the shipboard Beidou system^24^ and video surveillance to obtain the real-time position, speed and other characteristics of the ship. And real-time tracking, analysis and calculation of the ship’s estimated arrival time at the port.

**Figure 1:**
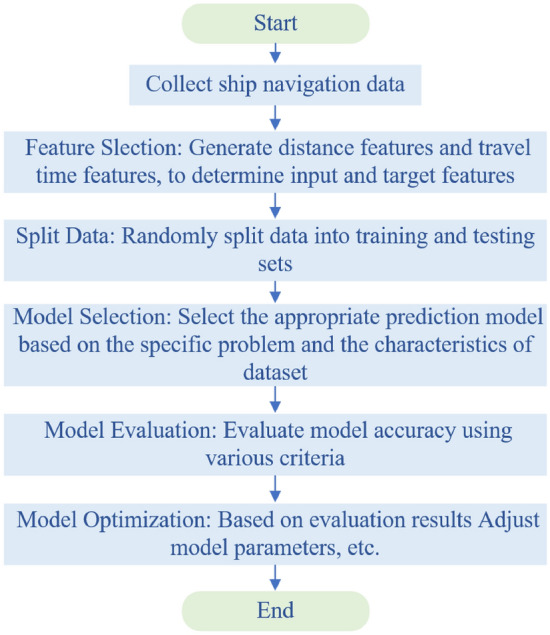
The flow chart for estimated time of the arrival solution.

### Predictive model

To obtain datasets of ship navigation on the Wujiang channel, we use the Beidou system^24^ to track ships and the planning data of ship passage. We collect the data every six minutes with timestamps. The characteristics of the data include ship identification number (i.e., *mmsi*), current latitude and longitude coordinates (i.e., *lat*, *lon*), speed (i.e., *speed*), ship type (i.e., $ship\_type$), origin, destination, destination latitude and longitude coordinates (i.e., $end\_lat$, $end\_lat$), the actual departure time, the actual arrival time, and whether reached the destination.

To make the predicted time more accurate, the coordinates under each timestamp are used to calculate the distance traveled by a ship. In order to facilitate the establishment and prediction of the model, we convert the timestamp in the data to date type. And subtract the departure time from the timestamp to get the time that the ship has traveled and convert the duration to seconds. In the process of dividing the data set, we randomly divided the training set and the test set, and the size $test\_size$ of the test set was selected as 0.08. Finally, train on historical data and make predictions by building a predictive model. The specific solution flow chart is shown in Fig. 1.

**Figure 2:**
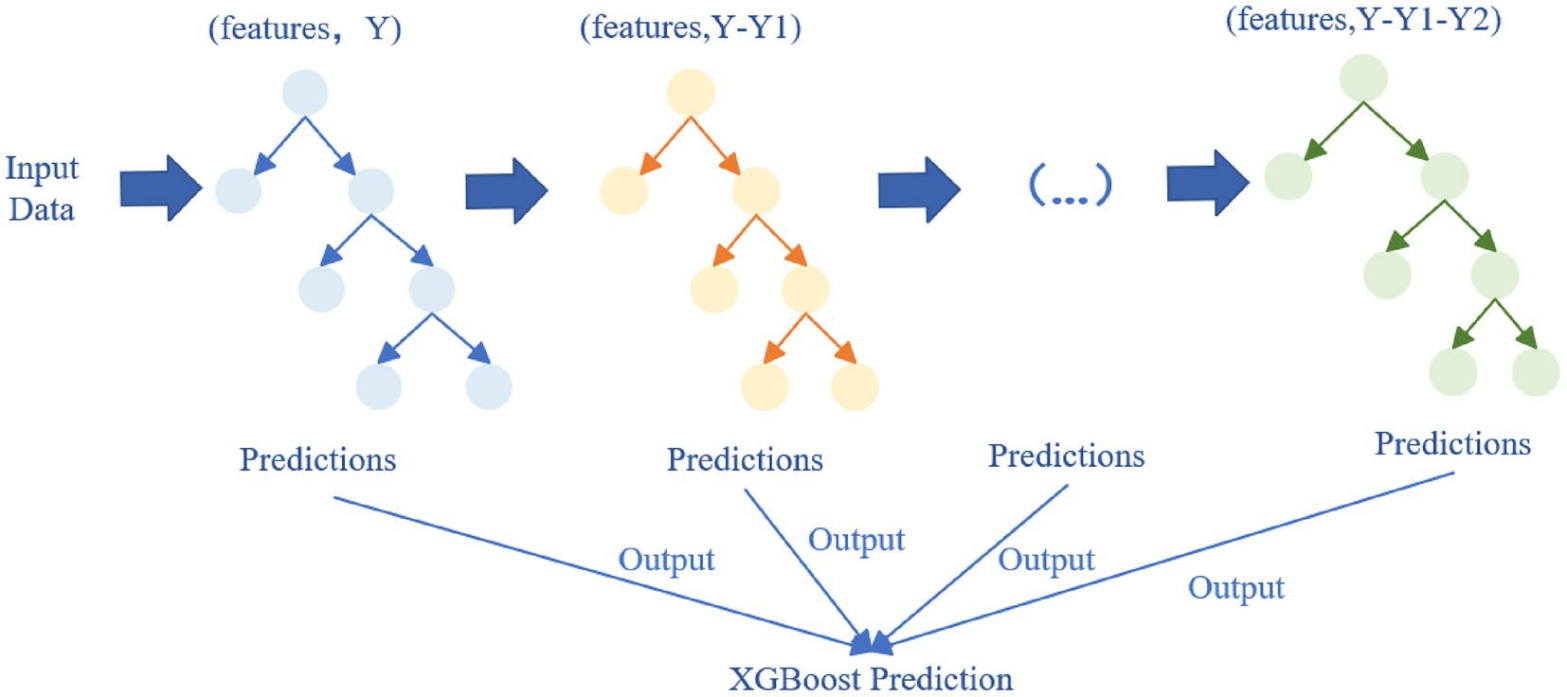
XGBoost regression prediction model.

The timestamp interval of adjacent data in the dataset that we collected is short, which can accurately grasp the various characteristics of the ship during its travel. During the solution process, the estimated ship arrival time is transformed into a regression prediction problem by utilizing the time stamp. Extreme Gradient Boosting (XGBoost)^25^ works efficient on small to medium datasets and can adaptively learn the weights of each decision tree. It has good generalization performance and is widely used in the field of data science. It can effectively solve the problem of predicting the expected arrival time of ships. So we choose the XGBoost model for prediction. XGBoost is an extreme boosting tree model based on the Boosting integrated learning algorithm, and an integrated gradient boosting algorithm based on a decision tree. Composed of many classification and regression trees (“weak learners”), the data set used by each regression tree is the entire data set, and the generation of each tree can be regarded as a single complete regression tree generation process. There is a sequence between trees. The results of each previous regression tree affect the prediction result of the next regression tree, that is, the latter regression tree is affected by the deviation of the previous regression tree during the prediction process. In each iteration, a new weak learners adjusts the model for the next iteration based on the difference between the previous model’s predictions and the true values. The core idea of Boosting is to sum the results of all weak classifiers to the predicted value, and then the next weak classifier fits the error function to the gradient of the predicted value (the error between the predicted value and the true value), thus continuously reducing the residuals until the error requirement of the system is satisfied. Figure 2 illustrates the process.

## The proposed real-time scheduling model

### Mathematical model

#### Analyze shipping efficiency

The shipping safety of the downstream navigation channel of the Silin Hydropower Station is mainly relevant to the water level change, flow rate, water level change rate, and downstream flow height. Among them, downstream water level and daily water level variation, and water flow rate have great correlation with discharge flow. The increase or decrease of the downstream flow affects the downstream water level and flow rate, while water level and downstream flow change rates are also related to the discharge flow. Therefore, the main factor affecting downstream channel safety is the discharge flow. Table 1 presents the specific factors that impact navigation safety.

**Table 1:** Factors affecting the efficiency of shipping.

Influence factors	Descriptions
Discharge flow	In order to meet the navigational conditions, the discharge flow needs to meet a certain range
Daily water level variation	The power station needs to perform grid peaking, but the downstream flow fluctuates, so the daily change in water level has to meet certain conditions
Hourly water level variation	Considering the safety of shipping, in addition to the daily variation of water levels, the condition of hourly variation should be satisfied
Downstream water level change rate	The rate of the changes of downstream water levels shall meet constraints
Water flow rate	The flow rate of water should meet certain requirements for ship passing
Downstream flow height	Downstream flow height affects the safety of ship navigation

**Figure 3:**
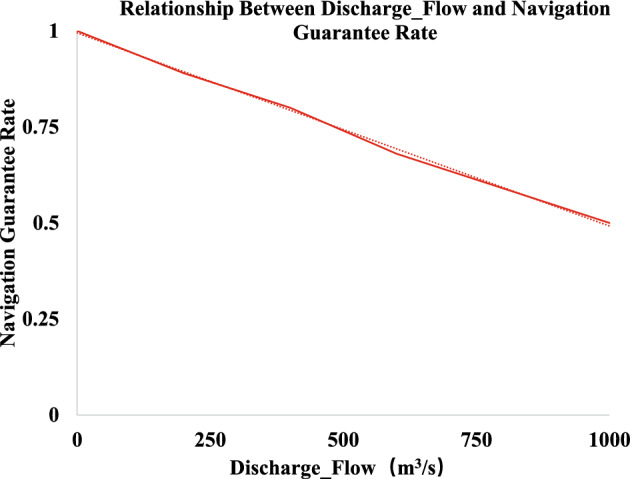
Relationship between discharge flow and navigation assurance rate.

Analyzing the historical data of the Silin Hydropower Station, the navigation assurance rate is more than 0.9 when the discharged flow rate of the Silin Hydropower Station is 0-200 m3/s; and more than 0.8 when it is 200–400 m$^3$/s. With the increase of the discharge flow, the navigation assurance rate decreases. The relationship between the specific Silin Hydropower Station discharge flow and the navigation assurance rate is shown in Fig. 3.

#### Constraints

The constraints of the Silin hydropower station scheduling system are illustrated in the section.

**Figure 4:**
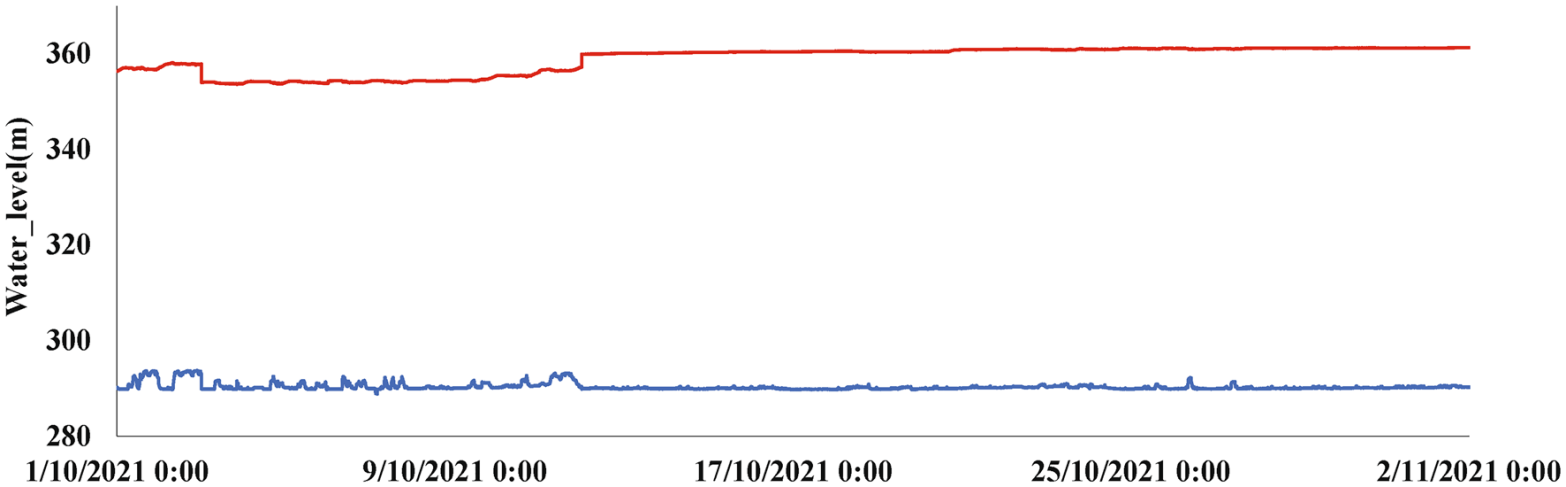
The trend of tailwater level and upstream water level.

Water level restriction in the operation of hydropower stations: in order to ensure the stability of hydropower station operations and the safety of downstream areas, we must impose restrictions on water level heights in the runtime of the stations. We thus have

1 $$\begin{aligned} Z_{\min }^j \le {Z^j} \le Z_{\max }^j, \end{aligned}$$

where $Z^{j}$ is the operating water level of the power stations at time *j*; $Z_{max}^{j}$ and $Z_{min}^{j}$ are the upper and lower limits of the operating water level of the power station at time *j*, respectively. The water level trend of the Silin Hydropower Station is shown in Fig. 4, in which the reddish brown line indicates the upper limit of water level operation of the Silin Hydropower Station and the blue line indicates the lower limit of water level operation of the Silin Hydropower Station.

**Figure 5:**
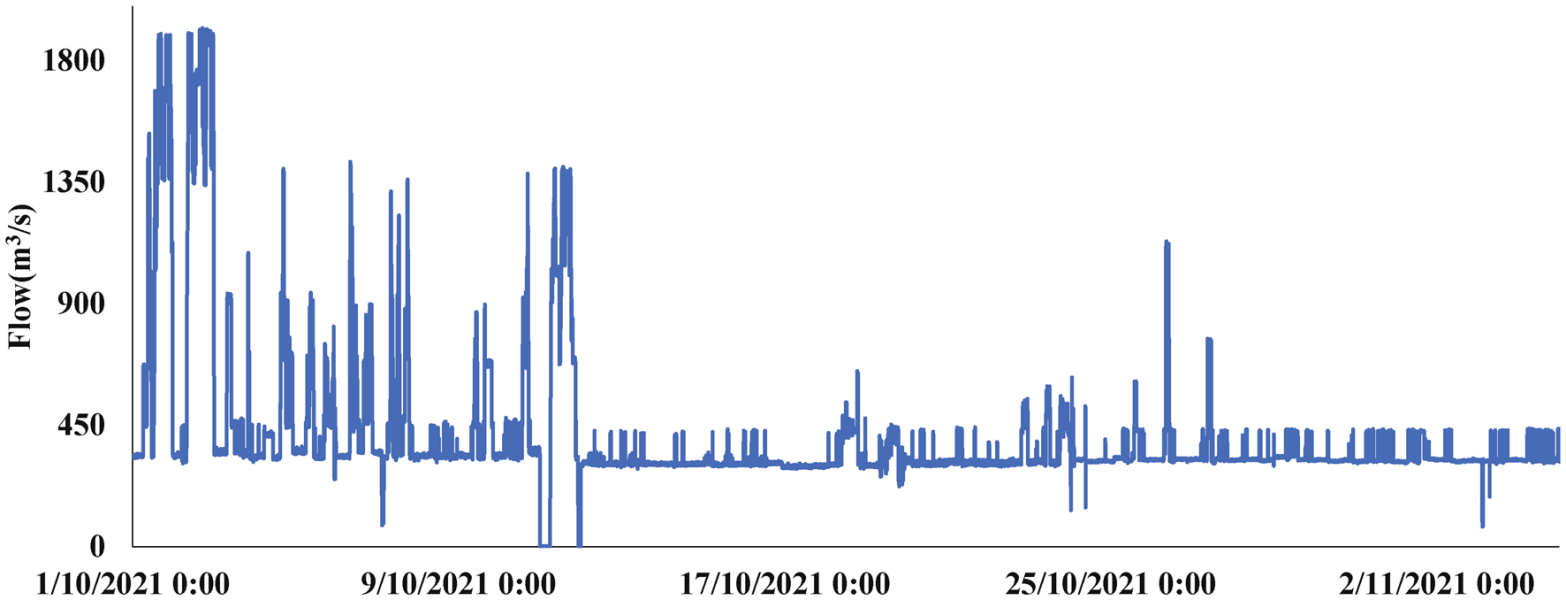
The flow trend of the Silin Hydropower Station.

Hydropower station discharge flow constraints: discharge flow and downstream navigation benefits and downstream ecological stability are closely related to the need to meet certain conditions.

2 $$\begin{aligned} Q_{\min }^j \le Q_{}^j \le Q_{\max }^j, \end{aligned}$$

where $Q^j$ is the discharge flow of the power station at time *j*; $Q_{\max }^j$ and $Q_{\min }^j$ are the maximum and minimum discharge flow of the power station to meet the shipping requirements at time *j*. The flow trend of the Silin Hydropower Station from October 1 to 7, 2021 is shown in Fig. 5.

Capacity constraints of hydropower stations: In order to protect hydropower units and maintain the stability of grid peaking, the capacity needs to be within a certain range.

3 $$\begin{aligned} P_{\min }^j \le P_{}^j \le P_{\max }^j, \end{aligned}$$

where $P_{\max }^j$ and $P_{\min }^j$ are the maximum and minimum allowable output of a station.

Downstream water level variation constraint: the downstream water level must not only meet the maximum and minimum water levels but also the value of water level variation per unit by time. We have

4 $$\begin{aligned}{} & {} \Delta {Z_{{\rm d}}} \le {Z_{\rm{{d\_max}}}}, \end{aligned}$$

5 $$\begin{aligned}{} & {} \Delta {Z_h} \le \Delta {Z_{h\_\max }}, \end{aligned}$$

6 $$\begin{aligned}{} & {} {Z_{15\min }} \le {Z_{15\min \_\max }}. \end{aligned}$$

In the equations, $Z_{\rm {d}}$, $Z_h$, and $Z_{15\min }$ are daily, hourly, and 15-minute variations of downstream water levels, respectively. $Z_{\rm{{d\_max}}}$ , $Z_{h\_\max }$ , $Z_{15\min \_\max }$ are the corresponding maximum values.

Ship passing restriction: When a ship passes during the scheduling period, the load is required not to change, so the discharge flow during this scheduling period should be consistent with the discharge flow at the previous scheduling time. At the same time, the water storage capacity plus power generation at this time should be greater than or equal to the grid load demand. Given $R_{\rm{{Storage Capacity}}}^t$ as the storage capacity at time *t*, $F_{\rm{{Hydroelectricity}}}^t$ as the power generation of the hydropower station at time *t*, and $F_{\rm{{Electricity demand}}}^t$ as the grid load demand at time *t*, we thus have

7 $$\begin{aligned} R_{\rm{{Storage Capacity}}}^t + F_{\rm{{Hydroelectricity}}}^t \ge F_{\rm{{Electricity demand}}}^t. \end{aligned}$$

Non-negative conditional constraint: all the above variables must be non-negative.

#### Objective function

In the study , we consider to store the power generated by hydropower stations to the grid, and to discharge the storage system whenever the power generated by hydropower stations cannot meet the demands of the grid. In order to maximize the benefits of the hydropower station, our objective is to generate power to meet the grid demands through the downstream flow. We also aim to reduce the negative impact on the ship navigation as the downstream flow might stop the shipping. Therefore, in this paper, we have two objectives, namely the power generation and the shipping scheduling.

Power generation scheduling goal: we aim to maximize the total stored energy at the end of the scheduling time period, and the total stored energy is accumulated from the stored energy within each time period. The amount of energy stored in each time period is the sum of the amount of power generated in the time period plus the amount of energy stored, and minus the load demand on the grid in the period. The power generation capacity of a hydropower station is related to the discharge flow, which is expressed by the “water to determine electricity” method, shown as follows.

The stored energy in the *t*th time period is:

8 $$\begin{aligned} {\mathrm{{F}}_t} = \left. {\left\{ {9.81{P^t}\Delta t + {R^{t - 1}} - F_{\rm{{Electricity demand}}}^t} \right. } \right\} , \end{aligned}$$

9 $$\begin{aligned} R_{}^{t - 1} = 9.81\sum \limits _{j = 1}^{t - 1} {(P_{}^j} \Delta t - F_{\rm{{Electricity demand}}}^j), \end{aligned}$$

where $P^t$ represents the output of the hydropower station at time *t*, $R^{t-1}$ represents the stored energy at $t-1$, and $\Delta t$ is the calculated time interval. $F_{\rm{{Electricity demand}}}^t$ represents the grid load demand at time *t*. The outgoing force in the *t*th time period is:

10 $$\begin{aligned} {P^t} = {H^t}{Q^t}, \end{aligned}$$

where $H^t$ and $Q^t$ are the power output, head and generation quoted flow of the power station at time *t*, respectively. We set the initial stored energy to zero and the generation scheduling objective function F1:

11 $$\begin{aligned} M\mathrm{{ax }}{\mathrm{{F}}_1} = \left. {\left\{ {9.81\sum \limits _{j = 1}^T {H_{}^jQ_{}^j} \Delta t - F_{\rm{{Electricity demand}}}^\mathrm{{j}}} \right. } \right\} . \end{aligned}$$

Shipping scheduling goal: The mapping relationship between the discharge flow of a hydrapower station and the navigation assurance rate is known, and the navigation assurance rate is considered as the evaluation index of the shipping efficiency of a channel, and the larger its value represents the better the shipping efficiency. The shipping scheduling function is as follows:

12 $$\begin{aligned} M\mathrm{{ax }}{F_2} = \frac{1}{T}\sum \limits _{j = 1}^T {{k_j}} \left( {{Q^j}} \right) , \end{aligned}$$

where, *F2* is the downstream channel navigation scheduling objective function; $k_j$ is the guaranteed rate of navigation in the *j*-th time period; $Q^j$ is the discharge flow of the hydropower station in the *j*th time period.

### NSBWO optimization algorithm

NSBWO is an algorithm that combines the BWO^26^ algorithm and the NSGA-II^27^ algorithm and is oriented to multi-objective optimal scheduling. When evaluating the population, fast non-dominated sorting and crowding degree calculation are used, BWO algorithm is used when generating the offspring population, and finally the elite strategy is introduced to screen the offspring to retain excellent individuals.

There are three main differences between the NSGA-II algorithm and the traditional genetic algorithm. The first point is to perform fast non-dominated sorting when selecting individuals, which reduces the time complexity of algorithm operation and improves computational efficiency. The second point is to adopt elitism. After the generation of offspring is merged with the parent generation to obtain a new population, a fast non-dominated sort is performed, which increases the probability of retaining an excellent population. The third point is that the method of calculating the crowding distance is used as a criterion for selecting the best among individuals of the same class, which ensures the diversity of the population.

The BWO algorithm simulates three behaviors of beluga whales: exploration, exploitation, and falling. The transition between the three behaviors depends on the balance factor $B_{\rm {f}}$ , which is defined as:

13 $$\begin{aligned} {B_{\rm {f}}} = {B_0}(1 - T(2{T_{\max }})), \end{aligned}$$

where *T* is the current number of iterations, $T_{\max }$ is the maximum number of iterations, and B0 varies randomly between (0,1) in each iteration when $B_{\rm {f}} > 0.5$ for the exploration phase and when $B_{\rm {f}}< 0.5$ for the exploitation phase. As *T* increases, the range of $B_{\rm {f}}$ decreases from (0,1) to (0,0.5), and the probability of the exploitation phase increases.

The location of the beluga whales during the exploration phase is updated as follows:

14 $$\begin{aligned}{} & {} X_{i,j}^{T + 1} = X_{i,{p_j}}^T + (X_{r,{p_1}}^T - X_{i,{p_j}}^T)(1 + {r_1})\sin (2\pi {r_2}),j = even. \end{aligned}$$

15 $$\begin{aligned}{} & {} X_{i,j}^{T + 1} = X_{i,{p_j}}^T + (X_{r,{p_1}}^T - X_{i,{p_j}}^T)(1 + {r_1})\cos (2\pi {r_2}),j = \mathrm{{odd}}. \end{aligned}$$

$X_{i,j}^{T + 1}$ is the new position of the *i*th beluga in the *j*th dimension, ${P_{\text{j}}}(j = 1,2,\ldots ,d)$ is a random integer chosen from the d-dimension, $X_{i,{p_j}}^T$ is the position of the ith beluga in the $p_j$ dimension, $X_{r,{p_1}}^T$ is the current positions of the 1st and *r*-th beluga, respectively (*r* is a random number), and the range of random operators used by $r_1$ and $r_2$ for the augmented exploration phase is (0, 1).

The exploitation phase location updates are as follows:

16 $$\begin{aligned} X_i^{T + 1} = {r_3}X_{best}^T - {r_4}X_i^T + {C_1}{L_F}(X_r^T - X_i^T), \end{aligned}$$

where $X_i^T$ and $X_r^T$ are the current position of the *i*th beluga and the *r*-th beluga, respectively. $X_i^{T + 1}$ is the new position of the *i*-th beluga, $X_{best}^T$ is the best position, $r_3$ and $r_4$ are random numbers between (0,1), and $C_1$ is the random jump intensity measuring the strength of the Levy flight. $L_F$ is the Levy flight function.

The falling stage locations are updated as follows:

17 $$\begin{aligned} X_i^{T + 1} = {r_5}X_i^T - {r_6}X_r^T + {r_7}X_{step}^{}, \end{aligned}$$

where $r_5$, $r_6$ and $r_7$ are random numbers between (0,1) and $X_{step}$ is the step size defined as:

18 $$\begin{aligned} X_{step} = ({u_b} - {l_b})\exp ( - {C_2}T/{T_{\max }}), \end{aligned}$$

where $C_2$ is the step factor associated with whale fall probability and population size, and $u_b$ and $l_b$ are the upper and lower bounds of the variables, respectively. The whale fall probability $W_f$ is calculated as a linear function:

19 $$\begin{aligned} W_f^{} = 0.1 - 0.05T/T_{\max }^{}. \end{aligned}$$

**Figure 6:**
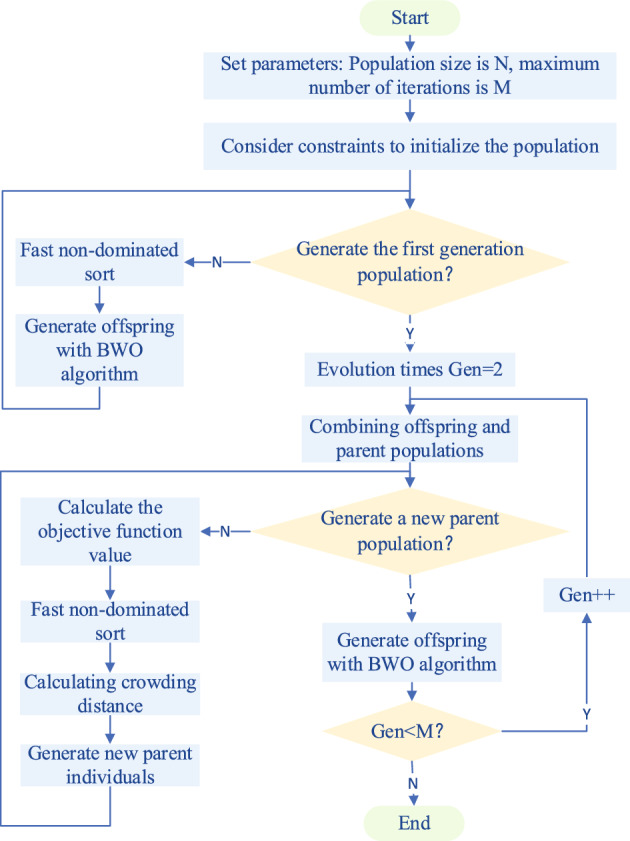
The flow chat of the NSBWO algorithm.

BWO is a population-based optimization algorithm, the inspired by the habits of whales in nature. It is updated according to its own position, food, and the positions of other belugas. Second, BWO can jump out of the local optimum by whale falling and introduces the Levy flight mechanism to improve convergence. The exploration capability of BWO is achieved by continuously expanding search agents throughout the search space, and the search trajectories are clustered around the global optimum achieving fast convergence. At the same time, the search history of BWO exhibits an approximately linear search pattern to avoid falling into local optima and ensure its global convergence. The results of BWO are closer to the global optimal solution than those of Genetic Algorithm (GA)^28^, Particle Swarm Optimization (PSO)^29^, Gray Wolf Algorithm (GWO)^30^, Gravitational Search Algorithm (GSA)^31^ in practical constrained optimization problems. Therefore, we introduce NSBWO based on BWO to optimize the scheduling of shipping and power generation effectiveness in solving the optimization problem of hydropower stations in this paper. The populations are screened using three methods in NSGA-II: fast non-dominated sorting, elite strategy, and crowding degree. Three stages of BWO exploration, exploitation, and falling are used to generate offspring subpopulations. Compared with NSGA-II, the global convergence is guaranteed and the number and quality of feasible solutions obtained by the algorithm are improved. The specific NSBWO algorithm flow is shown in Fig. 6.

### The model for hydropower stations

The whole scheduling model contains five parts: power load, expected arrival time of ships, constraint correction, energy storage strategy, and scheduling optimization. The five components complement each other to form a real-time multi-objective joint scheduling model. The implementation of the model is divided into eight steps as follows:

Step 1: Request grid load demand data from the grid system for the scheduling period and use a forecasting algorithm to predict the expected arrival times of passing ships.

Step 2: Code the downstream flow during the scheduling period with the number of coded bits equal to the number of scheduling times.

Step 3: Analyze the constraints that exist in the actual scheduling process.

Step 4: Determine if there is a ship passage at that moment, if not then scheduling the power generation normally, if there is a downstream flow need to be consistent with the previous period, if the power generation is not enough energy storage mechanism is needed to discharge.

Step 5: Initialize the Populations targets and perform non-dominated stratified ranking.

Step 6: Generate new offspring using the BWO algorithm.

Step 7: Perform fast non-dominated sorting and crowding distance calculation operations.

Step 8: Determine whether the number of iterations meets the condition, and if not, cycle the execution until the condition is met. The scheduling scheme with the optimal value of the discharge flow for each time period of the hydropower station is obtained.

**Figure 7:**
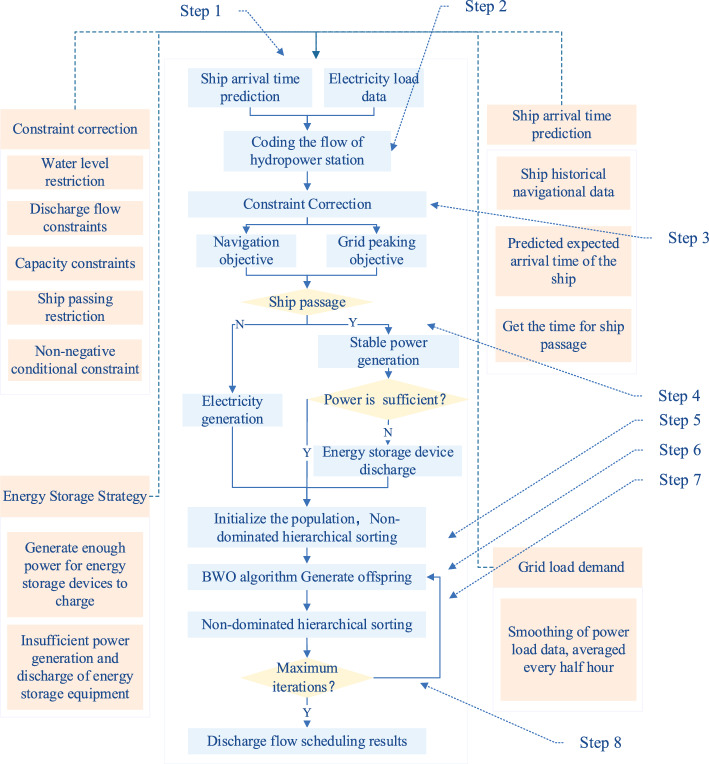
The multi-objective real-time scheduling model for hydropower stations.

The specific flow chart of the scheduling model is shown in Fig. 7.

## Experimental discussion

### Experiment introduction

The hardware and software environments used for the experiments are shown in Table 2. The hardware environment includes the processor, memory, and hard disk, and the software environment includes the operating system, programming environment, and development language.

**Table 2:** Experimental settings.

Environment	Configuration
Processor	12th Gen Intel(R) Core(TM) i7-12700H 2.70 GHz
RAM	DDR5 16G 4800MHZ
Hard disk	Samsung MZVL2512HCJQ-00BL2
OS	Windows11
Programming environment	Python3.9, MATLAB R2022a
Development language	Python, Matlab

**Table 3:** Navigation data samples (part I).

Row$\_$id	Ship$\_$type	Start$\_$lat	Start$\_$lon	End$\_$lat	End$\_$lon
1	1	104.830	26.592	108.192	27.807
2	1	104.830	26.592	108.192	27.807
3	1	104.830	26.592	108.192	27.807
...	...	...	...	...	...
197	1	104.830	26.592	108.192	27.807

**Table 4:** Navigation data samples (part II).

Row$\_$id	Lat	Lon	Speed (nmi/h)	Distance (km)	Duration (h)
1	104.830	26.592	4.780	0.000	00:00:00
2	104.852	26.593	5.610	2.150	00:05:19
3	104.869	26.594	3.960	4.307	00:10:52
...	...	...	...	...	...
197	108.192	27.807	0.000	287.952	16:22:53

#### Experiment purpose

Prediction of arrival time using the XGBoost model to obtain the actual arrival time of navigable ships. The XGBoost model is implemented to predict the arrival time and get the actual arrival time of navigable ships. Linear regression^32^, Ridge regression^33^ and CNN^34^ algorithms are also used for comparison. We implement the NSBWO algorithm for scheduling the discharge flow for each time period at the Silin Hydropower Station and analyze the feasibility of the method. And also use NSGA-II, GA-NSGA-II, reference vector guided evolutionary algorithm(RVEA)^35^ NSGA-III^36^, Multiobjective Evolutionary Algorithm Based on Decomposition(MOEA/D)^37^ algorithms for experimental comparison with the proposed algorithm.

### Experimental process

#### XGBoost test data processing

Table 3$\times$ Table 4 shows a sample of the navigation data of a cargo ship on the Wujiang Channel on October 1, 2021, which are seperated in two tables for the simplicity of reading. The data in the tables includes the attributes of ship type, latitude and longitude coordinates, speed, travel distance, and current travel time. Among the attributes, there exist three types of coordinates, namely the start (Start$\_$lat, Start$\_$lon), the desitination (End$\_$lat, End$\_$lon), and the current location (Lat, Lon). For the Ship$\_$type, we utilize the hot encoding method for discrete data types by setting the type of normal cargo ship to 1. The Duration and Distance are calculated based on the timestamp and coordinates.

#### XGBoost model experiment setup

In the process of predicting the expected arrival time of a ship, we select nine features, namely the latitude and longitude of the initial and final positions, the real-time latitude and longitude of the ship, the type of the ship, the speed of the ship, and the distance traveled by the ship, as input features, and the travel time of the ship as target features. The data is split into 92% training set and 8% test set. Use Eq. (20) to map the dataset to [0, 1] for normalization.

20 $$\begin{aligned} {X^*} = \frac{{X - {X_{Min}}}}{{{X_{Max}} - {X_{Min}}}}. \end{aligned}$$

Grid search is used during parameter tuning of XGBoost models. Perform a grid search on the maximum depth $max\_depth$ of the tree, the learning rate $learning\_rate$, and the maximum number of iterations $n\_estimators$. $max\_depth$ is set to [7, 9, 11, 13], $learning\_rate$ is set to [0.01, 0.05, 0.1], and $n\_estimators$ is set to [500, 1000]. Each parameter combination is traversed once, and the best parameter combination is finally obtained after comparison. The specific optimization parameters are listed in Table 5.

**Table 5:** Model parameter settings.

Parameter	Value	Description
n_estimators	500	Maximum number of iterations of the weak learner
learning_rate	0.05	Control the iteration rate to prevent over-fitting
gamma	0	The minimum descent value of the loss function required for node splitting, the larger the parameter value, the more conservative the algorithm
$max\_depth$	9	The maximum depth of the tree, the larger the value the more complex the model. Overfitting can be controlled by this value
alpha	1	L1 regularization term for the weights
lambda	0.5	L2 regularization term for the weights
subsample	1	Control the proportion of random sampling for each tree
booster	gbtree	Select base classifier, specify ascent model, commonly tree or linear model

#### Scheduling experiment data

The time interval between two adjacent rows of discharge flow data is one minute in the data set we obtained related to the Silin Hydropower Station. However, in the process of actual scheduling of hydropower stations, which is influenced by the start and stop of units, one minute scheduling is not in line with the actual scheduling situation. Therefore, we choose a scheduling interval of half an hour and smooth the data. The average value of the discharge flow is coded every half hour as the discharge flow in the scheduling period. The data related to the scheduling of the hydropower station are the lower limit of downstream flow (i.e., $Flow\_down$), the upper limit of downstream flow (i.e., $Flow\_up$), the grid load demand (i.e., *Load*), the ship passage (1 if there is a value otherwise 0), the guaranteed rate of navigation *k*, and the head *H*. The specific data is shown in Table 6.

**Table 6:** Scheduling data samples.

Flow$\_$down (m$^{3}$/s)	Flow$\_$up (m$^{3}$/s)	Load (kW)	Ship$\_$arrive	K	H
438.6	478.6	275.2	0	0.90	0.0625
650.6	690.6	391.6	0	0.82	0.0628
1182.7	1222.7	673.0	1	0.60	0.0622
1270.4	1310.4	716.2	0	0.58	0.0629
1075.9	1115.9	632.4	0	0.66	0.0627
...	...	...	...	...	...
1365.8	1405.8	775.0	0	0.54	0.0624

We selected the data between 8:00 and 18:00 on October 1, 2021 for the experiment. The estimated time of arrival of the ship obtained by using the XGBoost algorithm is shown in Table 7.

**Table 7:** Estimated arrival time samples.

Ship number	Ship$\_$Type	Direction of navigation	Source	Estimated arrival time
608	1	Down	APP	2021-10-01 10:08:16
605	1	Down	APP	2021-10-01 14:23:46
636	1	Down	AAP	2021-10-01 16:52:54

#### NSBWO model experiment setup

First, we set the parameters of the model. Since the objective function includes the maximum generation and the highest transportation effectiveness. So the number of objective functions is 2, the population size is set to 100, the maximum number of iterations is 200, and the crossover probability is 0.01, summarized in Table  8.

**Table 8:** Experimental variables.

Parameter	Value
Decision variables	20
Population size	100
Maximum iterations	250
Variation rate	1/20
Number of objective functions	2
Crossover probability	1%

**Figure 8:**
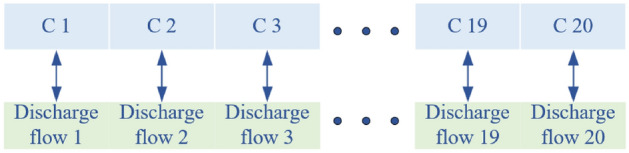
Decision variable coding.

The total time we schedule the discharge flow of the Silin Hydropower Station is from 8:00 to 18:00, and the scheduling time interval is that 30 min. We first encode the discharge flow, and the discharge flow in each scheduling time period corresponds to a code respectively. According to the time order, we can get the code segments of length 20, which are positioned as *C*1, *C*2,..., *C*20. We also set the upper and lower limits for each time period, as shown in Fig. 8.

The initial population is then initialized according to the constraints and flow restrictions for each time period to obtain the initial population and perform non-dominated ranking. Using BWO to generate offspring, the offspring population is merged with the parent population to obtain a new population. A new population is formed again by selecting the 100 best individuals in a non-dominated ranking of the new population. Finally, several iterations are performed until the maximum number of iterations is reached to obtain a solution for the discharge flow that takes into account both power generation and shipping.

### Result analysis

#### XGBoost experimental result analysis

**Figure 9:**
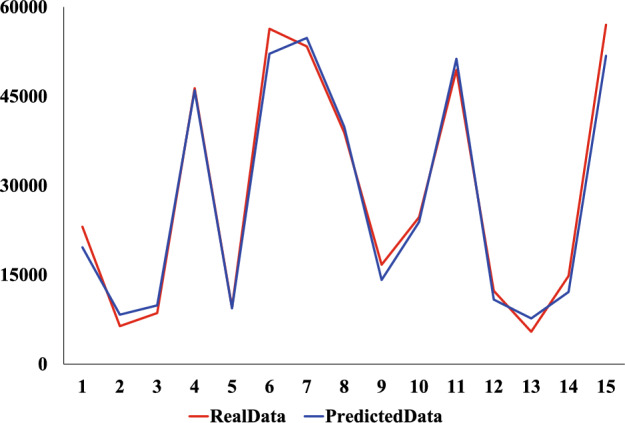
CNN predicted results.

**Figure 10:**
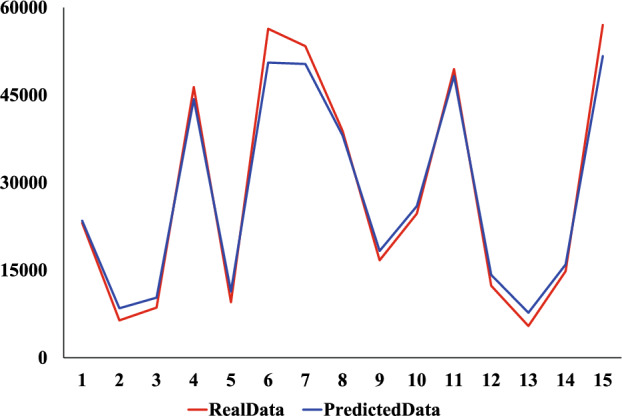
Ridge regression predicted results.

**Figure 11:**
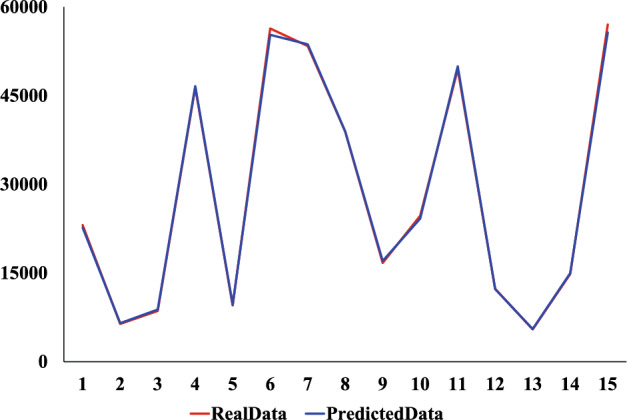
Linear regression predicted results.

**Figure 12:**
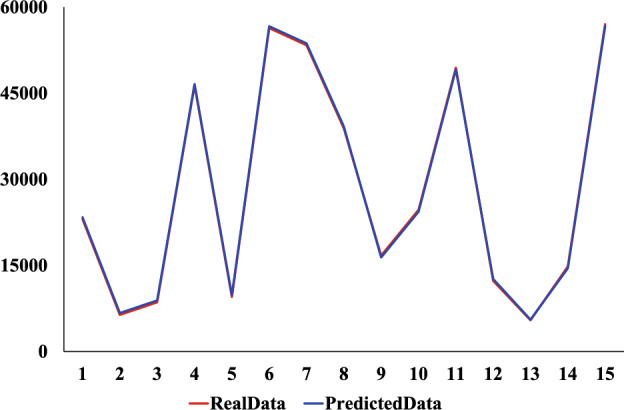
XGBoost predicted results.

We use the same dataset and divide it consistent with the XGBoost model. The comparison experiments were conducted using CNN, Ridge regression, and Linear regression models. The prediction results of the four models are shown in Figs. 9, 10, 11 and 12. Where the red curve represents the real data and the blue curve represents the predicted data. From the images, it can be concluded that the degree of XGboost fitting is close to Linear regression and slightly higher than CNN and Ridge regression.

After training the model using historical data, the test data is put into the trained model and the predicted values are calculated. The predicted values of the test data and their matched true values are used for model evaluation. In this paper, the evaluation metrics are evaluated by five regression models as shown in Table 9, where *n* denotes the number of samples, $y_{i}$ denotes the true value of the ith sample, $\widehat{y_{i} }$ denotes the predicted value of the ith sample, and $\overline{y}$ denotes the average of the true values.

**Table 9:** Evaluation metrics.

Performance metrics	Optimal value	Mathematical formula
MSE: Evaluates the degree of variability of the data. The smaller the value, the better the accuracy of the model	0	$MSE=\frac{1}{n} \sum _{i=1}^{T}\left( \widehat{y_{i} } - y_{i} \right) ^{2}$
MAE: The average distance between the model predicted value and the sample true value	0	$MAE = \frac{1}{n}\sum _{i=1}^{n} \left| \widehat{y_{i} } -y_{i}\right|$
RMSE: Evaluate the deviation between observed and actual values. The smaller the value, the better the prediction	0	$RMSE = \sqrt{\frac{1}{n}\sum _{i=1}^{n}(\widehat{y_{i} } - y_{i})^{2}}$
MAPE: Statistical indicator used to measure prediction accuracy, 0% indicates a perfect model, greater than 100% indicates a poor model	0	$MAPE = \frac{100\%}{n}\sum _{i=1}^{n} \left| \frac{\widehat{y_{i} }- y_{i}}{y_{i}}\right|$
$R^{2}$: A quantity used to measure how well the model fits. The scale interval is [0,1]. The closer to 1, the better the model fit	1	$R^{2} =1-\frac{\sum _{i=1}^{n}(\widehat{y_{i}} -y_{i} )^{2}}{\sum _{i=1}^{n}(y_{i}-\overline{y})^{2}}$

From the experiments, it is concluded that the XGBoost model is optimal in MSE^38^, MAE^39^, RMSE^40^, MAPE^41^, and R2^42^ metrics, with the R2 fitting metric reaching 0.983. followed by the Linear regression model outperforming the CNN and Ridge regression models. The specific metrics are shown in Table 10.

**Table 10:** Comparison of predictive models.

Model	MSE	MAE	RMSE	MAPE	$R^{2}$
CNN	73201	30	35	47.313	0.961
Linear regression	1958	7	6	0.064	0.980
Ridge regression	64110	28	33	0.153	0.966
XGBoost	1799	5	5	0.049	0.983

#### NSBWO experimental result analysis

**Figure 13:**
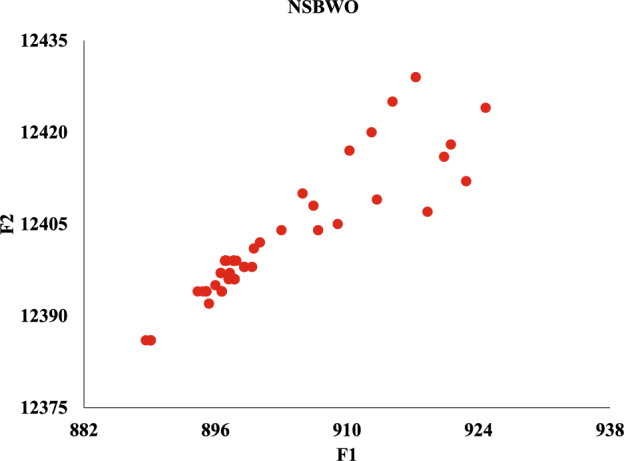
NSBWO scheduling.

We visualize the experimental results in Fig. 13. Each dot in the figure represents a population (i.e., feasible solution), the value of the discharge flow that satisfies the condition in each time period. The x axis represents the value of the solution’s objective function *F*1 and the y axis represents the value of the objective function *F*2. We aim to obtain solutions corresponding to both objective functions *F*1 and *F*2 that are as large as possible, so the closer the dot is to the upper right in Fig. 13 indicates the better quality of that solution set.

Figure 13 shows that the two solution sets with the objective function values of (917.321, 12429.1) and (924.751, 12424.1) have the highest fitness. Table 11 shows the discharge flow obtained from the solution set (924.751, 12424.1). The three periods in the table, 10:00-10:30, 14:00-14:30, and 16:30-17:00, have ships passing through the gate, and the flow rate under the unit that cannot be shut down needs to be consistent with the previous moment.

**Table 11:** The discharge flow obtained from the NSBWO experiments.

Time period	Discharge flow (m$^{3}$/s)	Time period	Discharge flow (m$^{3}$/s)
08:00–08:30	455.7	13:00–13:30	1374.0
08:30–09:00	650.6	13:30–14:00	1108.3
09:00–09:30	1182.7	14:00–14:30	1108.3
09:30–10:00	1310.4	14:30–15:00	1376.9
10:00–10:30	1310.4	15:00–15:30	1714.3
10:30–11:00	969.0	15:30–16:00	1840.0
11:00–11:30	485.8	16:00–16:30	1788.2
11:30–12:00	337.8	16:30–17:00	1788.2
12:00–12:30	330.9	17:00–17:30	1406.1
12:30–13:00	627.1	17:30–18:00	1365.8

**Figure 14:**
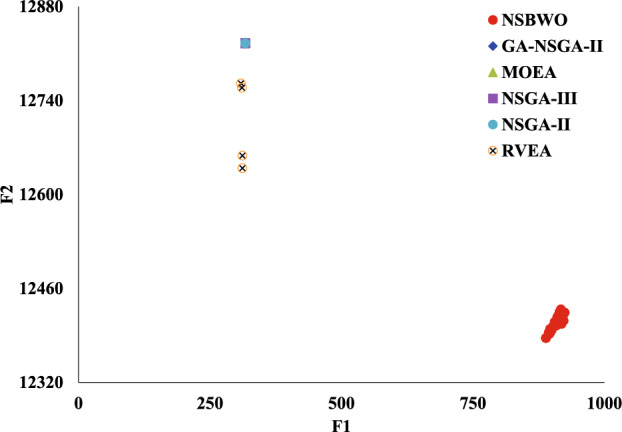
Scheduling algorithm comparison.

**Figure 15:**
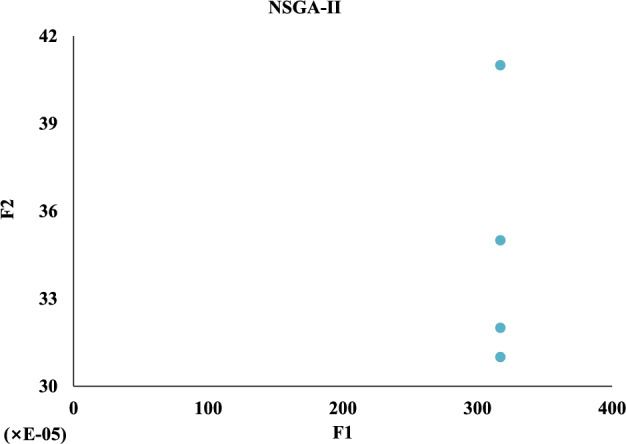
NSGA-II scheduling.

**Figure 16:**
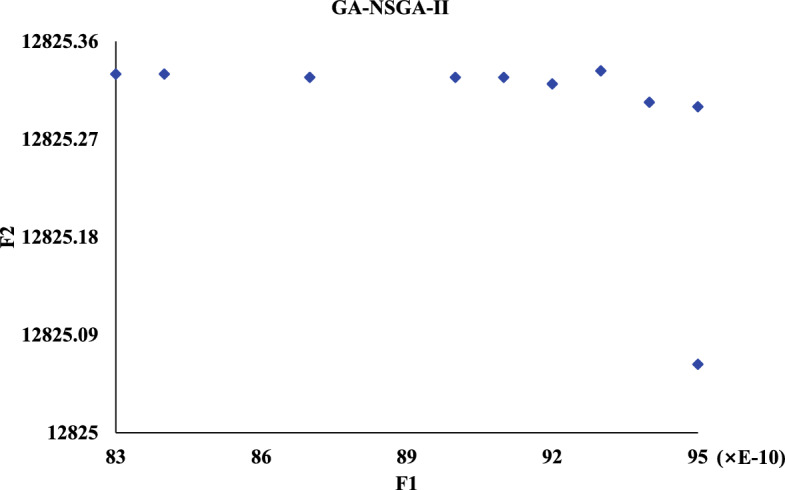
GA-NSGA-II scheduling.

**Figure 17:**
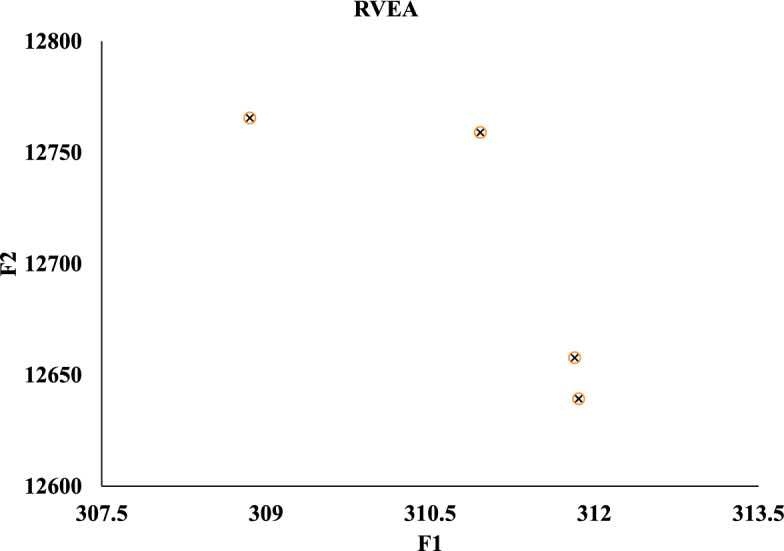
RVEA scheduling.

**Figure 18:**
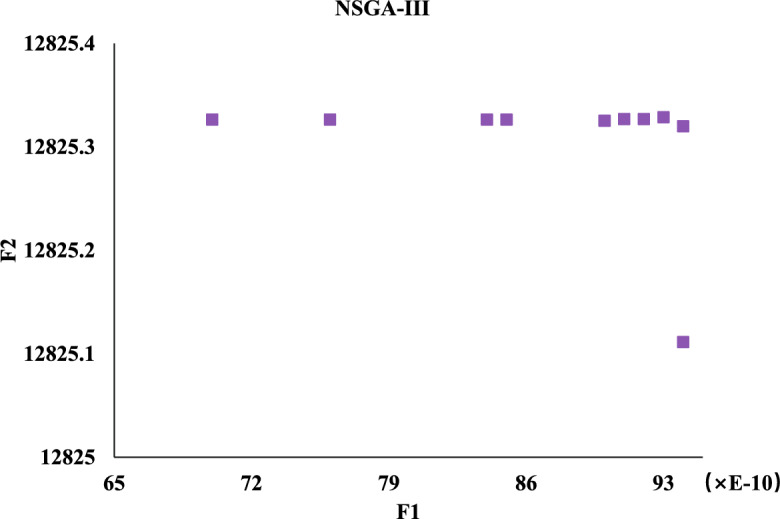
NSGA-III scheduling.

**Figure 19:**
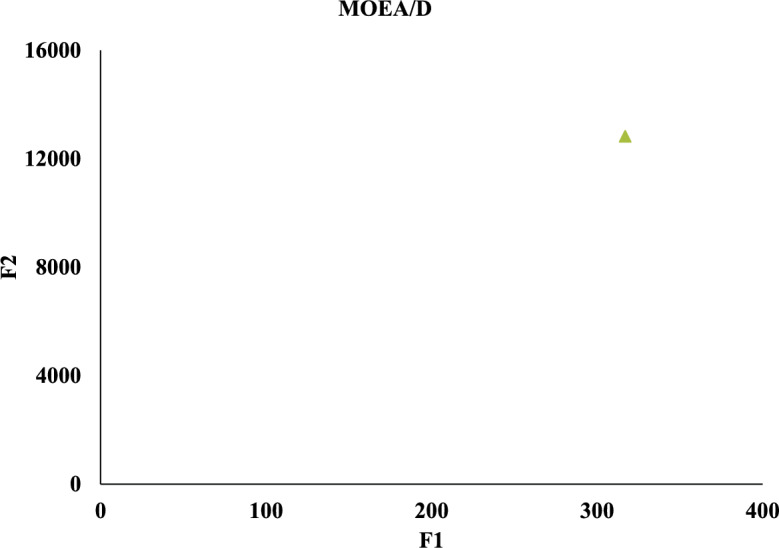
MOEA/D scheduling.

Meanwhile, we compare against five algorithms NSGA-II, GA-NSGA-II, RVEA, NSGA-III, and MOEA/D, and the results are in Fig. 14. It can be seen that the results of the solution objective function of NSBWO are the best. And the number of feasible solutions obtained by NSBWO scheduling is significantly larger than the other five optimization algorithms when the number of populations, constraints and maximum number of evolutionary generations are given the same. Since the results of the other methods are very close to each other, we individually show them in Figs. 15, 16, 17, 18 and 19 for better understanding of each method. To be noted, we normalize the real values in the figures for the visulization purpose. For the real values, each of the y values in Fig. 15 shall add 12825.326. Each of the x values in Fig. 16 shall add 316.93792304. Each of the x values in Fig. 18 shall add 316.93792304.

In each scheduling experiment, we choose the values of the objective functions *F*1 and *F*2 of the solution closest to the upper right corner of each figure for comparison. The results are in Table 12. We observe that the *F*2 value of NSBWO is slightly smaller than the average in the table, NSBWO can be considered to be the best as *F*1 is much larger than the average.

**Table 12:** Objective function comparison of scheduling models.

Scheduling algorithms	Objective function F1 value	Objective function F2 value
NSBWO	924.751	12424.000
GA-NSGA-II	316.877	12825.326
NSGA-II	316.938	12825.327
NSGA-III	316.938	12825.308
RVEA	313.698	12653.400
MOEA/D	316.940	12827.000

Next, we evaluate hypervolume (HV), spacing, and runtime metrics for the scheduling algorithms. The HV metric shows the convergence, stepwiseness, and cardinality of a learning set. If one solution set is better than another, its hypervolume index is greater. Spacing measures the standard deviation of the minimum distance of each solution to other solutions. The smaller the spacing value is, the more uniform the solution set is. The HV and spacing indicator formulas are shown in Eqs. (21) and (22).

21 $$\begin{aligned} S{\textrm{pacing}}({\textrm{P}}) = \sqrt{\frac{1}{{|P| - 1}}\sum \limits _{i = 1}^{|P|} {{{({\overline{d}} - {d_i})}^2}} }, \end{aligned}$$

where *P* denotes a set of uniformly distributed reference points sampled on PF, $d_i$ denotes the minimum distance between the *i*-th solution and other solutions in *P*, and ${\overline{d}}$ denotes the mean of all $d_i$.

22 $$\begin{aligned} HV = \delta \left( {\bigcup \nolimits _{i = 1}^{|S|} {{v_i}} } \right) , \end{aligned}$$

where $\delta$ stands for Lebesgue measure and it is used to measure volume, |*S*| represents the number of non-dominated solution sets, and $v_i$ represents the hypervolume formed by the reference point and the *i*th solution in the solution set.

The findings of the models are shown in Table 13. In terms of running time, MOEA/D has the longest running time, and the running times of other models are smaller and close. On HV, the NSBWO algorithm has the highest value, and the solution set obtained by NSBWO is significantly better than the solution set obtained by the other models. The Spacing of NSBWO is the highest, but because the other models obtain fewer solution sets, it does not mean that the solution set quality of NSBWO is lower than the other models.

**Table 13:** Evaluation of experimental results of scheduling models.

Scheduling algorithms	Execution time	HV	Spacing
NSBWO	0.175	0.076	7.252
GA-NSGA-II	0.152	$0.826\times 10^{-2}$	${0.262\times 10^{-5}}$
NSGA-II	0.144	${0.820\times 10^{-2}}$	${0.275\times 10^{-5}}$
NSGA-III	0.142	${0.828\times 10^{-2}}$	0.301
RVEA	0.111	${0.989\times 10^{-2}}$	0.917
MOEA/D	2.894	${0.826\times 10^{-2}}$	0.000

## Conclusion and future work

This paper focuses on the research of hydropower stations integrated into the power grid system, considering the functions of navigation and power generation. We propose a scheduling strategy that considers the real-time passage of ships and the use of energy storage to stabilize the power generation of hydropower stations. The strategy is applied to a real case of the Silin Hydropower Station on the Wujiang waterway in China to show the effectiveness of the proposed solution. However, the method can be applied in other places. Experiments have shown that the XGBoost algorithm predict the time when a ship passes through the gate in real time with $R^{2}$ to be 0.98, which is competitive compared with the other three popular models, namely CNN, Ridge Regression, and Linear Regression. The prediction method improves the waiting time for ships to pass through the lock and it also improves the power scheduling effectiveness of hydropower stations. When the power generation of a hydropower station is greater than the demand of the grid, the energy storage is ready to store energy. When it is less than the demand of the grid, the energy storage is discharged. This study uses the NSBWO algorithm combined with NSGA-II and BWO to optimize the discharge flow of the Silin Hydropower Station. This results in the daily operation plan of the hydropower station considering the dual factors of shipping and power generation.

For the future work, we would continue to study the multi-objective optimization problem of hydropower stations. We would also consider the real-time impact on the downstream ecological environment in the actual scheduling processes.

## Data Availability

The datasets used and/or analysed during the current study available from the corresponding author on reasonable request.
